# Prevalence of Nicotine Pouch Use Among Youth and Adults in Great Britain—Analysis of Cross-Sectional, Nationally Representative Surveys

**DOI:** 10.1093/ntr/ntae295

**Published:** 2025-01-10

**Authors:** Leonie Brose, Laura Bunce, Hazel Cheeseman

**Affiliations:** SPECTRUM Consortium, UK; Addictions Department, Institute of Psychiatry, Psychology and Neuroscience, King’s College London, London, UK; Action on Smoking and Health, London, UK; SPECTRUM Consortium, UK; Action on Smoking and Health, London, UK

## Abstract

**Background:**

The public health impact of new nicotine products will depend on their use by different population groups. We assessed the prevalence of nicotine pouch use among youth and adults in Great Britain (GB).

**Aims and Methods:**

Cross-sectional annual Action on Smoking and Health Smokefree GB Adult Surveys 2020-2024 (*n*: 12 247 to 13 266, 18+) and Action on Smoking and Health Smokefree GB Youth Survey 2024 (*n* = 2872 11-18-year-olds). Weighted proportions and 95% confidence intervals for pouch ever and current use among adults over time, and in 2024 among youth and adults overall, by socio-demographics, mental health, tobacco smoking, vaping, past-12-month gambling, cannabis, and alcohol use; for youth also family members’ smoking and vaping. Multivariable logistic regression assessed the association with ever pouch use.

**Results:**

The prevalence of adult ever and current use doubled from 2020 to 2024, reaching 5.4% (95% confidence interval = 5.0 to 5.8) and 1.0% (0.8-1.1). Among youth, 3.3% (2.7-4.0) reported ever use, including 1.2% (0.8-1.6) reporting current use. Ever use was associated with the use of other addictive products. Among adults, ever use was also more common among younger groups (18-24, 25-34, 35-44 vs. 55+), males, and those in rented accommodation or receiving mental health treatment. Among youth, ever use was also more common among those in London than elsewhere in England.

**Conclusions:**

Pouch use in GB is rare with about 1 in 100 youth and adults reporting current use. However, use appears to be increasing and is higher in some groups, including younger adults, males and people with experience of vaping, smoking, and use of other addictive products.

**Implications:**

While currently low, close monitoring of pouch use is indicated. It is currently concentrated among those with experience of nicotine use. However, given the higher levels of use among young adults and teenagers, consideration of regulation may be required to minimize uptake among groups that would otherwise not have used nicotine-containing products.

## Introduction

Nicotine pouches are small sachets designed to be placed in the mouth between the upper lips and gums to release nicotine. Nicotine pouches do not contain tobacco, although they are similar in appearance and use to snus, which contains tobacco and are sometimes mistakenly called snus (eg, see literature^1–3^).

For many countries, nicotine pouches do not fit neatly into existing regulations^4^ and for example in Great Britain (GB), they fall outside the regulation for tobacco and related products, as they do not contain tobacco, and outside of medical regulation, as no medicinal claims are made.^5^ Currently, there is no minimum age of sale or restrictions on packaging or marketing. Evidence, mainly from the United States, indicates that sales and marketing efforts have increased in the last few years.^6–10^ In 2024, the United Kingdom government introduced a Bill that included further regulations for these novel nicotine products including an age of sale.^11^ Due to a snap election, the Bill did not pass into law but is being bought back by the new government.^12^ It is therefore likely that products will be further regulated soon.

Public health effects depend on the health effects of a product and the number of people experiencing positive and negative health effects.^13^ For people who smoke, switching to nicotine pouches would be expected to have positive health effects,^7^ as smoking-related morbidity and mortality are caused primarily by the results of combustion, not nicotine.^14^ However, nicotine is the primary addictive component in smoking,^15^ raising concerns about increased nicotine pouch use among young people and people who have not used other nicotine products, particularly with some pouches able to deliver high doses of nicotine very quickly.^16^

Prevalence data, particularly from outside the United States, are scarce. A 2019 survey in the United Kingdom among adults who smoked and/or vaped or had done so until recently found that 4.4% had ever used pouches, including 2.7% currently using them; ever use was more common among younger adults, those living in London, with higher education and currently smoking and vaping.^17^ Population-level survey data from 2020 to 2021 showed that use among adults in GB was rare at just 0.3% but had doubled within a year.^18^ A more recent report described similar levels of overall use for 2023.^19^ Use was more common among men, younger adults, and those who smoked or had smoked, used nicotine replacement therapy, or vaped.^18,19^ Prevalence among adults may have changed since these survey data were collected and no prevalence data for youth have been published.

### Aims

This study used population-level surveys from GB to:

1. Describe the change in prevalence of ever and current use of nicotine pouches in adults over time;
2. Estimate prevalence of ever and current use of nicotine pouches among youth and adults in 2024;
3. Assess cross-sectional associations between ever use of nicotine pouches and: socio-demographics, mental health, use of or exposure to other nicotine-containing products, and use of other addictive products.

## Methods

### Design

The data provided in this survey study were obtained from cross-sectional online surveys conducted annually in February or March: the Action on Smoking and Health Smokefree GB Youth Survey 2024 (11-18-year-olds) and the Action on Smoking and Health Smokefree GB Adult Survey 2020 to 2024 (18 and over). Participants were drawn from existing online panels maintained by YouGov. Active sampling was used, which dynamically evaluates what surveys are available for a particular panel member based on their personal characteristics, and restrictions are put in place to ensure that only those who are selected from their panel of registered members are allowed to take part in the survey. For the youth survey, informed consent was provided either by the parents of those aged 11 to 15 years or by individuals aged 16 to 18 years. For the adult survey, informed consent was provided by participants. The surveys are designed to be representative of the population, and survey weights are derived by YouGov to make the data representative across age, sex and region for youth and age, sex, social class, region, level of education, and votes at the previous election for adults. Ethical approval for the analyses was not required because this study involved a secondary analysis of preexisting anonymized data.

### Measures

The full wording of the questions and response options including recoding is available in the preregistration (https://osf.io/p9gmv). Inclusion of don’t know and prefer not to say response options varied across the survey; these are mentioned below where included.

#### Pouch Use

Participants in the adult survey were asked the following question: Nicotine pouches are non-medicinal consumer pouches of nicotine, which are placed in the mouth and sucked. Brands include Lyft, Skruf, Zin, Nordic Spirit, and Velo [or 2020 and 2021: Zin, Nordic Spirit, and Velo]. Which of the following statements BEST applies to you?

a) I have never heard of nicotine pouches and have never tried them;
b) I have heard of nicotine pouches but have never tried them;
c) I have tried nicotine pouches but do not use them (anymore);
d) I have tried nicotine pouches and still use them;
e) Don’t know.

Youth were asked a slightly simplified version of the question: Nicotine pouches are pouches that contain nicotine, but not tobacco. Brands include Lyft, Skruf, Zin, Nordic Spirit, and Velo. Which of the following statements BEST applies to you? The response options were identical except for (e) which was “Don’t want to say.”

This was coded into two versions of the measure: (1) for ever pouch use, responses c and d were coded yes, all others as no; (2) for current pouch use, response d was coded as yes, all others as no. This measure was first included in the adult survey in 2020 and in the youth survey in 2024.

#### Adult Survey

For the adult survey, socio-demographics included *Age* (18-24, 25-34, 35-44, 45-54, 55+), *Sex* (female, male), *Ethnicity* (White, Asian, Black, Other/mixed which included prefer not to say), and *Region* (England excluding London, London, Scotland, Wales). *Occupational grade* was grouped into ABC1 and C2DE based on five categories according to the National Readership Survey classification^20^: AB (higher and intermediate managerial, administrative, and professional); C1 (supervisory, clerical and junior managerial, administrative, and professional); C2 (skilled manual workers); D (semi-skilled and unskilled manual workers); or E (receiving only the state pension, casual and lowest grade workers, unemployed with state benefits only). *Education* (low: no certifications/GCSEs and equivalents, medium: A-levels and equivalents/technical qualifications below degree or high: university degree or above) and *Housing tenure* (Own—outright, with a mortgage and shared ownership, Rent—private landlord, Rent—local authority/housing association, Neither/Other) were also included.

*Mental health (treatment)* was recorded as yes, no, or unknown (for prefer not to say or don’t know) by asking respondents (1) whether in the last 12 months they had any treatment or taken any medication for any of a list of conditions and (2) whether they were on the waiting list for any mental health treatment.

The use of several other products was measured. *Smoking status* was recorded as never (“I have never smoked”), past (“I used to smoke but have given up now”), or current (“I smoke but I don’t smoke every day” or “I smoke every day”) smoking. The question preamble specified tobacco smoking. *Vaping status* was also recorded as never [“I have never heard of vapes (e-cigarettes) and have never tried them” or “I have heard of vapes (e-cigarettes) but have never tried them”], past [“I have tried vapes (e-cigarettes) but do not use them (anymore)”], current [“I have tried vapes (e-cigarettes) and still use them”], or unknown [Don’t know]. *Cannabis use* in the past 12 months was recorded as yes, no, or unknown, *Gambling* in the past 12 months was recorded as yes or no, and *Alcohol use* was recorded using British versions of the AUDIT-C questions.^21,22^ All adults were asked: “How often do you have a drink containing alcohol? Never (scored 0), Monthly or less (1), 2–4 times per month (2), 2–3 times per week (3), 4 or more times per week (4).” Those who ever drink alcohol were asked two more questions: (1) “How many units of alcohol do you drink on a typical day when you are drinking? 0 to 2 (0), 3 to 4 (1), 5 to 6 (2), 7 to 9 (3), 10 or more (4)”; (2) “How often have you had more than 6 units on a single occasion in the last year? Never (0), Less than monthly (1), Monthly (2), Weekly (3), Daily or almost daily (4).” A definition of units was provided as were prefer not to say and don’t know options. Responses were coded into low risk (scores <5), higher risk (scores 5 and above), and unknown.

#### Youth Survey

For youth, socio-demographics included *Age* (11-15, 16-18); *Sex* (male, female); *Region* (England excluding London, London, Scotland, or Wales), and *Family occupational grade* (ABC1, C2DE, unknown).^20^  *Ethnicity* was not asked.

*Mental health* was measured using two ratings about how happy/anxious they felt yesterday on a scale from 0 to 10 and coded into low (0 to 6), high (7 to 10), or unsure happiness and low (0 to 5), high (6 to 10), or unsure anxiety.^23^

Youth were asked to select all family members that smoked or vaped; these were coded into *Smoking in family* (yes, no, unsure) and *Vaping in Family* (yes, no, unsure). The respondent’s own *Smoking status* was recorded as never, past, current, or unknown based on the question: “Which ONE of the following BEST applies to you? a) I have never smoked cigarettes, not even a puff or two; b) I have only ever tried smoking cigarettes once; c) I used to smoke sometimes but I never smoke cigarettes now; d) I sometimes smoke cigarettes now but less than one a week; e) I usually smoke between one and six cigarettes a week; f) I usually smoke more than six cigarettes a week; g) Don’t want to say.” Response a was coded as never smoked, responses b and c as tried in the past, responses d to f as currently smoking, g as unknown. *Vaping status* was recorded as never, past, current, or unknown based on two questions: 1. “Have you heard of vapes, also called electronic cigarettes or e-cigarettes? a) Yes, I have; b) No, I haven’t; c) Don’t know.” All who responded (a) were then asked the following question: 2. “Which ONE of the following is closest to describing your experience of vapes (e-cigarettes)? a) I have never used vapes (e-cigarettes); b) I have only tried vapes (e-cigarettes) once or twice; c) I use vapes (e-cigarettes) sometimes, but no more than once a month; d) I use vapes (e-cigarettes) more than once a month, but less than once a week; e) I use vapes (e-cigarettes) more than once a week but not every day; f) I use vapes (e-cigarettes) every day; g) I used vapes (e-cigarettes) in the past but no longer do; h) Don’t want to say.” Those who responded b to question 1 or a to question 2 were coded as never vaped, those who responded b or g to question 2 as past, those who respond c to f as currently vaping, and those who responded c to question 1 or h to question 2 as unknown. *Cannabis use, Gambling*, and *Alcohol use* in the past 12 months were each coded as yes, no, unknown (for prefer not to say).

### Sample

The adult survey was completed by *n* = 12 809 in 2020, *n* = 12 247 in 2021, *n* = 13 088 in 2022, *n* = 12 271 in 2023, and *n* = 13 266 in 2024; the youth survey 2024 was completed by *n* = 2872 respondents. The full samples were included for aim 1 and aim 2. For aim 3, where less than 5% were unsure or unable to give a response for a variable, they were excluded from analysis; where 5% or more fell into this category, they were retained as a separate category (see changes from preregistration), leaving *n* = 12 462 adults and *n* = 2584 youth for analysis.

### Analysis

Analysis used weighted data and used SPSS version 27 and R.

1. Proportion and 95% confidence intervals (CIs) for pouch ever use and current use among all adults in 2020, 2021, 2022, 2023, and 2024.
2. Proportion and 95% CIs for pouch ever use and pouch current use among youth and among adults overall and by socio-demographics, mental health, smoking, vaping, past-12-month cannabis use, gambling, and alcohol use. For youth, smoking and vaping in the family were also included.
3. Logistic regression with ever use as outcome, bivariate (unadjusted) for each covariate listed, followed by multivariable (adjusted) models that included those variables significantly associated with the outcome in the bivariate analyses (and only one of socio-economic status, education, housing tenure).

### Changes From Preregistration

The region was split into four rather than the planned three groups, as previous evidence has indicated higher prevalence specifically in London. For youth, cannabis use was only asked of those aged 16 to 18 and therefore excluded from the adjusted analysis for aim 3. An additional post hoc sensitivity analysis restricted to the 16 to 18 age group assessed associations between cannabis use and ever pouch use adjusting for the same variables as the main analysis (excluding age group).

We also changed the way we treated those who did not know or preferred not to give a response. For many questions, such as those around new products, these are valid responses. We therefore kept them as separate categories for the sample description and overall prevalence figures; for aims 2 and 3, we excluded the category from analysis if applicable to less than 5% of respondents.

We did not calculate a prevalence ratio for the change from 2020 to 2024, as this did not offer additional information.

## Results

Socio-demographics, mental health, smoking, vaping, cannabis use, gambling, and alcohol use among adults are described in Table 1 for adults and Table 2 for youth.

**Table 1.** Sample Description Adults in Great Britain, 2024 (Weighted Data, Unweighted *n* = 13 266)

Characteristic	Groupings	*n*	% (95% CI)
**Age group**	**18-24**	1618	12.1 (11.6-12.6)
	**25-34**	2170	16.3 (15.6-16.9)
	**35-44**	2329	17.5 (16.8-18.1)
	**45-54**	2072	15.5 (14.9-16.2)
	**55+**	5150	38.6 (37.8-39.5)
**Sex**	**Male**	6506	48.8 (47.9-49.6)
	**Female**	6833	51.2 (50.4-52.1)
**Ethnicity**	**White**	11 150	83.6 (83.0-84.2)
	**Asian**	688	5.2 (4.8-5.5)
	**Black**	349	2.6 (2.4-2.9)
	**Other/mixed**	1153	8.6 (8.2-9.1)
**Region**	**England (excl London)**	9645	72.3 (71.5-73.1)
	**London**	1857	13.9 (13.3-14.5)
	**Wales**	674	5.1 (4.8-5.4)
	**Scotland**	1163	8.7 (8.2-9.2)
**Occupational grade** ^a^	**ABC1**	7142	53.5 (52.7-54.4)
	**C2DE**	6197	46.5 (45.6-47.3)
**Education**	**Low**	3695	27.7 (26.9-28.5)
	**Medium**	5563	41.7 (40.8-42.6)
	**High**	4081	30.6 (29.8-31.4)
**Housing tenure**	**Own**	7686	57.6 (56.8-58.5)
	**Rent, private**	2325	17.4 (16.8-18.1)
	**Rent, social**	1536	11.5 (11.0-12.1)
	**Other**	1792	13.4 (12.9-14.0)
**Mental health treatment**	**No**	9037	67.8 (66.9-68.6)
	**Yes**	3131	23.5 (22.7-24.2)
	**Unknown**	1170	8.8 (8.3-9.3)
**Smoking**	**Never**	7157	53.7 (52.8-54.5)
	**Past**	4458	33.4 (32.6-34.3)
	**Current**	1724	12.9 (12.4-13.5)
**Vaping**	**Never**	9636	72.2 (71.5-73.0)
	**Past**	1989	14.9 (14.3-15.5)
	**Current**	1427	10.7 (10.2-11.3)
	**Unknown**	287	2.1 (1.9–2.4)
**Cannabis use**	**No**	11 777	88.3 (87.7-88.8)
	**Yes**	977	7.3 (6.9-7.8)
	**Unknown**	585	4.4 (4.1-4.8)
**Gambling**	**No**	6827	51.2 (50.3-52.1)
	**Yes**	6511	48.8 (47.9-49.7)
**Alcohol use**	**Low risk**	7568	56.7 (55.9-57.6)
	**Higher risk**	4220	31.6 (30.8-32.5)
	**Unknown**	1551	11.6 (11.1-12.2)

^a^ABC1 includes high and intermediate managerial, administrative, or professional, supervisory, clerical, and junior managerial, administrative or professional occupations; C2DE includes skilled manual workers, semi and unskilled manual workers, state pensioners, casual or lowest grade workers, unemployed with state benefits only.

**Table 2.** Sample Description Youth in Great Britain, 2024 (Weighted Data, Unweighted *n* = 2872)

Characteristic	Grouping	*n*	% (95% CI)
**Age group**	**11-15**	1784	62.1 (60.1-64.1)
	**16-18**	1088	37.9 (35.9-39.9)
**Sex**	**Male**	1473	51.3 (49.1-53.5)
	**Female**	1399	48.7 (46.5-50.9)
**Region**	**England (excl London)**	2118	73.8 (71.9-75.5)
	**London**	389	13.6 (12.1-15.2)
	**Wales**	138	4.8 (4.02-5.75)
	**Scotland**	226	7.9 (7.2-8.6)
**Family occupational grade** ^a^	**ABC1**	1991	69.3 (67.2-71.3)
	**C2DE**	868	30.2 (28.2-32.3
	**Unknown**	13	0.4 (0.2-0.8)
**Smoking in family**	**No**	1846	64.3 (62.1-66.3)
	**Yes**	926	32.2 (30.2-34.3)
	**Unknown**	101	3.5 (2.8-4.3)
**Vaping in family**	**No**	1641	57.1 (55.0-59.3)
	**Yes**	841	29.3 (27.3-31.3)
	**Unknown**	390	13.6 (12.1-15.1)
**Happiness**	**Low/medium**	881	30.7 (28.7-32.7)
	**High/very high**	1885	65.6 (63.6-67.7)
	**Unknown**	106	3.7 (2.9-4.6)
**Anxiety**	**Low/medium**	1700	59.2 (57.1-61.3)
	**High**	981	34.2 (32.1-36.2)
	**Unknown**	191	6.6 (5.6-7.58)
**Smoking**	**Never**	2251	78.4 (76.6-80.1)
	**Past**	394	13.7 (12.3-15.2)
	**Current**	168	5.8 (4.9-6.9)
	**Unknown**	59	2.0 (1.5-2.8)
**Vaping**	**Never**	2225	77.5 (75.7-79.2)
	**Past**	334	11.6 (10.4-13.0)
	**Current**	243	8.5 (7.4-9.7)
	**Unknown**	70	2.4 (1.8-3.2)
**Cannabis use** ^b^	**No**	915	84.1 (81.6-86.4)
	**Yes**	120	11.0 (9.1-13.2)
	**Unknown**	53	4.9 (3.6-6.5)
**Gambling**	**No**	2256	78.5 (76.7-80.3)
	**Yes**	560	19.5 (17.8-21.3)
	**Unknown**	57	2.0 (1.5-2.7)
**Alcohol use**	**No**	1532	53.3 (51.2 - −55.5)
	**Yes**	1247	43.4 (41.3-45.5)
	**Unknown**	93	3.3 (2.5-4.2)

^a^ABC1 includes high and intermediate managerial, administrative, or professional, supervisory, clerical, and junior managerial, administrative or professional occupations, C2DE includes skilled manual workers, semi and unskilled manual workers, state pensioners, casual or lowest grade workers, unemployed with state benefits only.

^b^Only asked of 16-18-year-olds, unweighted *n* = 1228.

### Prevalence of Ever and Current Use Among Adults 2020-2024

**Figure 1.**
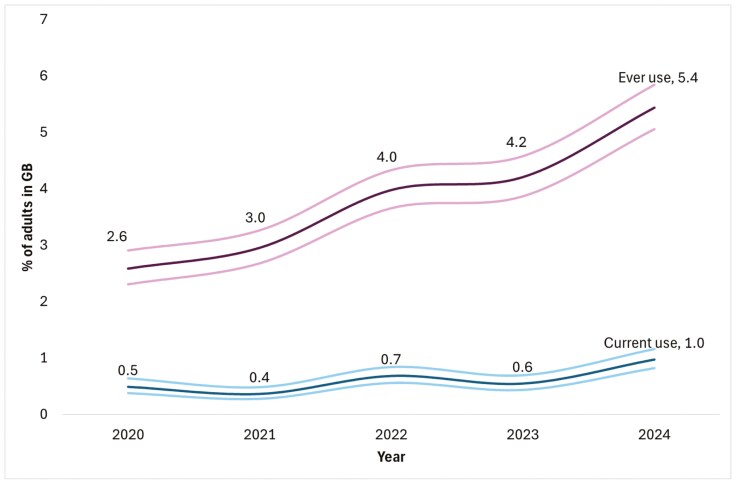
Prevalence of ever and current use among adults in Great Britain from 2020 to 2024.

Prevalence of ever and current use had both doubled from 2020 to 2024, albeit at very low levels, reaching 5.4% (95% CI = 5.0 to 5.8) and 1.0% (95% CI = 0.8 to 1.1) respectively in 2024 (Figure 1).

Lighter lines indicate 95% CIs. Axis truncated due to the low percentages shown.

### Prevalence of Ever and Current Pouch Use in 2024

#### Adults

In 2024, 40.2% of adults had never heard of nicotine pouches, a further 50.9% had heard of them but never tried them, and 3.5% responded don’t know, while 4.4% had tried them but did not use them anymore and 1.0% had tried them and were currently using them (combining to 5.4% ever use). Prevalence among groups of the population varied (Table 3). Ever use was low among those who had never smoked (1.5%), never vaped (1.9%), or were aged 55 and over (2.7%); it was high among people who had used cannabis in the past 12 months (22.0%), were currently vaping (17.8%), or currently smoking (16.8%). Current use was low among those aged 55 and over (0.2%), those who had never vaped (0.3%), female respondents (0.4%), and high among those groups that also reported high ever use, that is, people who had used cannabis in the past 12 months (5.6%) were currently vaping (4.1%) or currently smoking (3.3%, Table 3).

**Table 3.** Nicotine Pouch Use Among Adults in Great Britain, 2024, Weighted Data

		Current use	Ever use^a^	Ever use^a^		Ever use^a^	
		% (95% CI)	% (95% CI)	Unadjusted odds ratio (95% CI)	*p*-value	Adjusted odds ratio (95% CI)	*p*-value
**Overall**		0.97 (0.81-1.14)	5.42 (5.04-5.81)				
**Age group**	**18-24**	2.53 (1.76-3.29)	9.31 (7.89-10.72)	**3.72 (2.89-4.78)**	**<.001**	**2.95 (2.11-4.12)**	**<.001**
	**25-34**	1.77 (1.21-2.32)	8.43 (7.27-9.60)	**3.44 (2.71-4.35)**	**<.001**	**1.88 (1.42-2.50)**	**<.001**
	**35-44**	1.24 (0.79-1.68)	6.05 (5.08-7.02)	**2.34 (1.82-3.00)**	**<.001**	**1.34 (1.01-1.76)**	**.04**
	**45-54**	0.65 (0.31-1.00)	5.17 (4.22-6.13)	**1.95 (1.49-2.55)**	**<.001**	1.27 (0.95-1.69)	.11
	**55+**	0.15 (0.05-0.26)	2.67 (2.23-3.12)	Ref		Ref	
**Sex**	**Male**	1.57 (1.27-1.87)	7.12 (6.49-7.74)	**1.92 (1.63-2.27)**	**<.001**	**1.62 (1.35-1.94)**	**<.001**
	**Female**	0.40 (0.25-0.55)	3.75 (3.30-4.20)	Ref		Ref	
**Ethnicity**	**White**	0.85 (0.68-1.02)	5.17 (4.76-5.58)	Ref			
	**Asian**	1.03 (0.28-1.79)	5.51 (3.81-7.22)	1.05 (0.73-1.52)	.80	1.19 (0.79-1.80)	.40
	**Black**	1.63 (0.30-2.96)	7.45 (4.69-10.20)	1.53 (0.99-2.35)	.06	1.49 (0.91-2.42)	.11
	**Other/mixed**	1.85 (1.07-2.63)	6.89 (5.43-8.35)	**1.41 (1.09-1.83)**	**.01**	0.97 (0.72-1.31)	.84
**Region**	**England (excl London)**	0.69 (0.53-0.86)	4.90 (4.47-5.33)	Ref			
	**London**	1.84 (1.23-2.45)	7.58 (6.38-8.79)	**1.61 (1.31-1.98)**	**<.001**	1.10 (0.87-1.41)	.43
	**Wales**	1.81 (0.81-2.82)	5.54 (3.82-7.27)	1.11 (0.77-1.59)	.58	1.05 (0.71-1.55)	.80
	**Scotland**	1.38 (0.71-2.05)	5.93 (4.58-7.29)	1.22 (0.93-1.61)	.15	1.16 (0.86-1.57)	.32
**Occupational grade** ^b^	**ABC1**	1.00 (0.77-1.23)	5.40 (4.88-5.93)	Ref		Not included	
	**C2DE**	0.94 (0.70-1.18)	5.39 (4.82-5.95)	1.00 (0.86-1.17)	.97		
**Education**	**Low**	0.82 (0.53-1.11)	5.11 (4.40-5.82)	Ref		Not included	
	**Medium**	1.07 (0.80-1.34)	5.71 (5.10-6.32)	1.19 (0.98-1.45)	.08		
	**High**	0.98 (0.67-1.28)	5.22 (4.54-5.90)	1.03 (0.83-1.27)	.80		
**Housing tenure**	**Own**	0.57 (0.40-0.74)	3.79 (3.36-4.21)	Ref		Ref	
	**Rent, private**	1.52 (1.02-2.01)	8.33 (7.21-9.46)	**2.33 (1.91-2.84)**	**<.001**	**1.27 (1.01-1.60)**	**.04**
	**Rent, social**	1.17 (0.63-1.71)	8.23 (6.85-9.60)	**2.30 (1.84-2.88)**	**<.001**	**1.29 (1.00-1.66)**	**.05**
	**Other**	1.80 (1.18-2.41)	6.05 (4.95-7.16)	**1.60 (1.25-2.04)**	**<.001**	0.97 (0.72-1.30)	.82
**Mental health treatment**	**No**	0.53 (0.40-0.70)	3.70 (3.33-4.12)	Ref			
	**Yes**	1.97 (1.48-2.46)	9.51 (8.48-10.54)	**2.73 (2.33- 3.21)**	**<.001**	**1.66 (1.37–2.00)**	**<.001**
	**Unknown**	1.70 (1.10–2.62)	7.44 (6.04–9.12)	**2.09 (1.64–2.67)**	**<.001**	**1.40 (1.03–1.90)**	**.03**
**Smoking**	**Never**	0.50 (0.33-0.66)	1.54 (1.26-1.83)	Ref		Ref	
	**Past**	0.81 (0.54-1.07)	7.16 (6.40-7.92)	**5.78 (4.54-7.36)**	**<.001**	**4.17 (3.17-5.50)**	**<.001**
	**Current**	3.34 (2.50-4.19)	16.82 (15.05-18.58)	**15.68 (12.23-20.11)**	**<.001**	**5.01 (3.71-6.76)**	**<.001**
**Vaping**	**Never**	0.30 (0.19-0.41)	1.90 (1.63-2.17)	Ref		Ref	
	**Past**	1.80 (1.22-2.38)	13.65 (12.14-15.16)	**8.23 (6.72-10.07)**	**<.001**	**3.16 (2.51-3.98)**	**<.001**
	**Current**	4.13 (3.10-5.17)	17.76 (15.78-19.74)	**11.36 (9.24-13.97)**	**<.001**	**3.63 (2.85-4.63)**	**<.001**
**Cannabis use**	**No**	0.50 (0.37-0.63)	3.76 (3.42-4.10)	Ref		Ref	
	**Yes**	5.58 (4.14-7.02)	22.02 (19.42-24.62)	**7.31 (6.11-8.75)**	**<.001**	**2.03 (1.64-2.51)**	**<.001**
**Gambling**	**No**	0.62 (0.44-0.81)	4.29 (3.81-4.78)	Ref		Ref	
	**Yes**	1.33 (1.05-1.61)	6.55 (5.95-7.15)	**1.57 (1.33-1.84)**	**<.001**	**1.25 (1.04-1.49)**	**.02**
**Alcohol use**	**Low risk**	0.48 (0.32-0.63)	3.55 (3.13-3.97)	Ref		Ref	
	**Higher risk**	1.71 (1.32-2.10)	8.45 (7.61-9.29)	**2.58 (2.18-3.06)**	**<.001**	**1.43 (1.18-1.72)**	**<.001**
	**Unknown**	1.76 (1.39-2.21)	6.28 (5.17–7.61)	**1.66 (1.26-2.18)**	**<.001**	0.96 (0.72-1.30)	.80

Unweighted *n* = 13 266 for proportions, *n* = 12 462 for regressions as Unknown category excluded if applicable to < 5% of respondents. Results in bold text indicate confidence intervals not including 1.

^a^Includes current use.

^b^ABC1 includes high and intermediate managerial, administrative, or professional, supervisory, clerical, and junior managerial, administrative or professional occupations, C2DE includes skilled manual workers, semi and unskilled manual workers, state pensioners, casual or lowest grade workers, unemployed with state benefits only.

#### Youth

Among youth, 56.6% had never heard of nicotine pouches, a further 37.8 had heard of them but never tried them, 2.3% did not want to say, while 2.1% had tried them and did not use them anymore and 1.2% had tried them and were currently using them (3.3% ever use). High levels of ever use were reported by those who had used cannabis in the past 12 months (26.6%), currently smoked (24.5%), or currently vaped (22.1%), whereas low levels were reported among those who had never smoked (0.8%), never vaped (1.1%), or not had alcohol in the past 12 months (1.5%, Table 4). Similarly, current use was high among those currently smoking (12.5%) or vaping (7.1%) and those who had used cannabis in the past 12 months (5.5%), while low rates were reported among those with no family members vaping, from occupational grades C2DE, and those who had never smoked or had no family members smoking (all 0.4%, Table 4).

**Table 4.** Nicotine Pouch Use Among Youth in Great Britain, 2024, Weighted Data

		Current use	Ever use^a^	Ever use^a^		Ever use^a^	
		% (95% CI)	% (95% CI)	Unadjusted odds ratio (95% CI)	*p*-value	Adjusted odds ratio (95% CI)	*p*-value
**Overall**		1.22 (0.82-1.62)	3.32 (2.67-3.98)				
**Age group**	**11-15**	1.07 (0.59-1.55)	2.27 (1.58-2.96)	Ref		Ref	
	**16-18**	1.47 (0.76-2.19)	5.06 (3.76-6.36)	**2.05 (1.32-3.18)**	**.001**	0.93 (0.53-1.62)	.79
**Sex**	**Male**	1.48 (0.86-2.10)	4.02 (3.01-5.02)	**1.73 (1.10-2.74)**	**.02**	**1.98 (1.16-3.37)**	**.01**
	**Female**	0.95 (0.44-1.46)	2.59 (1.76-3.43)	Ref		Ref	
**Region**	**England (excl London)**	0.84 (0.45-1.23)	2.62 (1.94-3.30)	Ref		Ref	
	**London**	3.53 (1.70-5.36)	7.53 (4.91-10.15)	**3.44 (2.11-5.59)**	**<.001**	**3.62 (1.98-6.62)**	**<.001**
	**Wales**	1.27 (0-3.14)	2.90 (0.10-5.70)	0.89 (0.27-2.96)	.85	1.62 (0.43-6.10)	.48
	**Scotland**	0.81 (0-1.99)	2.95 (0.74-5.15)	1.03 (0.41-2.56)	.95	1.57 (0.58-4.28)	.38
**Family** **occupational grade** ^b^	**ABC1**	1.48 (0.95-2.01)	3.40 (2.61-4.20)	Ref		Ref	
	**C2DE**	0.41 (0-0.83)	2.95 (1.83-4.08)	0.79 (0.48-1.31)	.36	0.82 (0.46-1.46)	.50
**Smoking in family**	**No**	0.44 (0.14-0.74)	2.26 (1.59-2.94)	Ref		Ref	
	**Yes**	2.34 (1.37-3.32)	5.22 (3.79-6.65)	**2.25 (1.45-3.49)**	**<.001**	0.77 (0.44-1.37)	.38
**Vaping in family**	**No**	0.38 (0.08-0.68)	1.54 (0.94-2.13)	Ref		Ref	
	**Yes**	2.15 (1.17-3.13)	5.94 (4.34-7.53)	**3.86 (2.34-6.37)**	**<.001**	1.62 (0.85-3.09)	.14
	**Unknown**	2.76 (1.14-4.39)	5.22 (3.01-7.42)	**3.53 (1.78-7.03)**	**<.001**	**4.64 (2.00-10.80)**	**<.001**
**Happiness**	**Low/medium**	0.81 (0.22-1.40)	4.49 (3.12-5.86)	**1.66 (1.06-2.58)**	**.03**	1.28 (0.74-2.20)	.38
	**High**	1.28 (0.77-1.78)	2.69 (1.96-3.41)	Ref		Ref	
**Anxiety**	**Low/medium**	1.18 (0.67-1.69)	2.54 (1.79-3.29)	Ref		Ref	
	**High**	1.10 (0.45-1.76)	4.63 (3.31-5.94)	**1.77 (1.14-2.75)**	**.01**	1.11 (0.66-1.87)	.70
	**Unknown**	2.19 (0.11-4.27)	3.61 (0.96-6.26)	0.60 (0.11-3.36)	.56	1.02 (0.16-6.58)	.99
**Smoking**	**Never**	0.44 (0.17-0.72)	0.89 (0.50-1.28)	Ref		Ref	
	**Past**	1.05 (0.04-2.05)	8.72 (5.93-11.50)	**12.39 (6.79-22.61)**	**<.001**	**4.63 (2.16-9.95)**	**<.001**
	**Current**	12.49 (7.49-17.49)	24.46 (17.95-30.96)	**39.06 (20.92-72.91)**	**<.001**	**6.94 (3.00-16.03)**	**<.001**
**Vaping**	**Never**	0.57 (0.25-0.88)	1.11 (0.67-1.54)	Ref		Ref	
	**Past**	0.90 (0-1.91)	3.64 (1.63-5.65)	**3.15 (1.49-6.68)**	**.003**	1.67 (0.67-4.17)	.28
	**Current**	7.07 (3.85-10.29)	22.11 (16.89-27.32)	**26.87 (15.93-45.32)**	**<.001**	**9.27 (4.01-21.42)**	**<.001**
**Cannabis use** ^c^	**No**	0.70 (0.16-1.24)	2.07 (1.15-2.99)	Ref		Not included	
	**Yes**	5.56 (1.45-9.67)	26.58 (18.66-34.50)	**17.26 (8.92-33.42)**	**<.001**		
**Gambling**	**No**	0.72 (0.37-1.06)	2.32 (1.70-2.95)	Ref		Ref	
	**Yes**	3.24 (1.77-4.71)	7.32 (5.16-9.48)	**3.45 (2.21-5.39)**	**<.001**	1.19 (0.68-2.07)	.55
**Alcohol use**	**No**	0.73 (0.30-1.15)	1.51 (0.90-2.12)	Ref		Ref	
	**Yes**	1.80 (1.07-2.54)	5.47 (4.21-6.73)	**4.34 (2.57-7.33)**	**<.001**	1.46 (0.74-2.85)	.27

Unweighted *n* = 2872 for proportions, *n* = 2505 for regressions as Unknown category excluded if applicable to < 5% of respondents. Results in bold text indicate confidence intervals not including 1.

^a^Includes current use.

^b^ABC1 includes high and intermediate managerial, administrative, or professional, supervisory, clerical, and junior managerial, administrative or professional occupations, C2DE includes skilled manual workers, semi and unskilled manual workers, state pensioners, casual or lowest grade workers, unemployed with state benefits only.

^c^Only asked of 16-18-year-olds, unweighted *n* = 1228.

### Association of Ever Use With Other Characteristics and Behaviors

#### Adults

Pouch use was associated with many other characteristics and behaviors among adults. In adjusted analysis accounting for socio-economics, mental health, and smoking and vaping simultaneously (Table 3), each age group under 55 was more likely to have ever used pouches than the oldest age group, and men were more likely to have used them than women. Compared with people who owned their home, people who rented from local authorities/housing associations or privately were more likely to have used pouches. Adults who reported mental health treatment and those with unknown mental health treatment status were more likely to have used pouches. There were clear associations with the use of other addictive products. Those who currently smoked or had smoked in the past were more likely to also have used pouches; similarly, those who had vaped or were currently vaping were more likely to have used them than those who had never vaped. Having used cannabis or gambled in the past 12 months was also associated with pouch use as was higher risk alcohol use (Table 3).

Some additional associations were significant only in unadjusted analysis. Adults categorized as other and mixed ethnicities were more likely to have used pouches than respondents categorized as White and adults living in London were more likely to have used pouches than those who lived in other regions of England. Compared with people who owned their homes, those having other housing tenure (not owning or renting) were more likely to have used pouches (Table 3).

Occupational grade and education were not significantly associated with ever use (Table 3).

#### Youth

Among youth, pouch ever use was also associated with other characteristics and behaviors. In analysis adjusting for other characteristics (Table 4), it was more likely among male respondents and those living in London. Being unsure about vaping in the family was associated with pouch use. Ever use was strongly associated with past or current smoking, current vaping and was also more likely among those who had gambled or used alcohol in the past 12 months (Table 4).

Only in unadjusted analysis, age, smoking in the family, known vaping in the family, happiness, anxiety, past vaping, gambling, or alcohol use in the past 12 months were associated with ever pouch use. Pouch ever use was strongly associated with past-12-month cannabis use in the unadjusted analysis, due to the restriction of the question to the older age group, cannabis use was not included in the adjusted analysis. However, a post hoc sensitivity analysis restricted to the 16 to 18 age group found that cannabis use remained associated in adjusted analysis (odds ratio = 3.83, 95% CI = 1.63 to 9.01, *p* = .002). For smoking, vaping, and cannabis use, the odds ratios, particularly the unadjusted ones, are extremely large with extremely wide CIs, indicating that results should be treated with some caution (Table 4).

Family occupational grade was not associated with pouch use (Table 4).

## Discussion

In 2024, among adults in GB, current and ever use of nicotine pouches was rare at 1.0% and 5.4%, respectively, despite a noticeable increase since 2020. This is the first study reporting on the prevalence of nicotine pouch use among youth, with just more than 3% reporting ever and 1% current use in 2024, providing a useful baseline for future monitoring efforts. The ever use of nicotine pouches was closely associated with the consumption of other nicotine products and appears linked with cannabis use both among adults and youth and more common among males in both groups. Among adults, it was also associated with younger age, social housing, receiving or waiting for treatment for mental health problems, gambling, and riskier use of alcohol. Among youth, those living in London (vs. other areas of England) were also more likely to have ever used pouches.

The present findings are consistent with earlier reports^17–19^ in that pouch use was more common among younger adults, those smoking and vaping, and those living in London. Our findings extend previous findings of more use among male adults to adolescents. We did not find clear evidence that pouch use was associated with education or occupation; however, any differences may currently be less apparent due to overall very low prevalences.

The study’s strengths lie in the use of very large, population-wide surveys that are enhanced by weighting and cover the full age range of the population from as young as 11 years old. As any survey study, it relies on recall and self-reporting, which can introduce biases and inaccuracies. There is also the possibility of misunderstandings regarding different types of products; the description of how pouches are used will be improved for future surveys. However, the more detailed question assessing pouch use on its own rather than alongside other products will have strengthened reliability.^24^ People not providing a clear answer about their nicotine pouch use were categorized as never users, which may have underestimated the levels of ever and current use. Furthermore, the overall low prevalence of nicotine pouch use and the large differences between groups make it challenging to generate reliable estimates, particularly for groupings associated with relatively large differences in use. Some associations found among adults were not found among youth, this may be due to the much smaller sample of youth. Some measures were blunt, for example, mental health status was not assessed using a validated screener or diagnostic instrument, and different measures were used for adults and youth. However, the brief measures allowed use in large population surveys. It is possible that the coronavirus disease 2019 (COVID-19) pandemic affected prevalence trends over time among adults, particularly in 2021 when “lockdowns” were in place during the time of the survey.^25^

The findings highlight the need for ongoing monitoring to track trends in nicotine pouch use. Additionally, they add to the evidence of why regulation should be considered. Higher levels of use in young and disadvantaged populations provide further case for action to regulate these products. While use as an alternative to smoking would reduce the public health harms from smoking, public health benefits will also be seen from a properly regulated market, which avoids use among people who would otherwise not have used any nicotine and teenagers.

Evidence on the effects of nicotine pouch use on smoking^26^ or on the use of other nicotine-containing products is currently very limited. Research into this and the effects of nicotine pouches on addiction or health outcomes will further elucidate the public health effects of nicotine pouches.

## Conclusion

Pouch use in GB is currently low among adults and youth with about 1 in 100 people reporting current use. However, use appears to be increasing and is much higher in some groups, including younger adults, males and people with experience of vaping, smoking, and use of other addictive products. Monitoring and consideration of regulations are required.

## Data Availability

For access to the data, please contact the authors. Codes for recoding and SPSS analysis are available at the Open Science Framework https://osf.io/p9gmv.
